# Consensus-Based Formation Control with Time Synchronization for a Decentralized Group of Mobile Robots

**DOI:** 10.3390/s24123717

**Published:** 2024-06-07

**Authors:** Michał Siwek

**Affiliations:** Faculty of Mechatronics, Armament and Aerospace, Military University of Technology, Kaliskiego 2 Street, 00-908 Warsaw, Poland; michal.siwek@wat.edu.pl

**Keywords:** mobile robot, consensus tracking, formation control, time synchronization, synchronous control, decentralized group of robots

## Abstract

The development and study of an optimal control method for the problem of controlling the formation of a group of mobile robots is still a current and popular theme of work. However, there are few works that take into account the issues of time synchronization of units in a decentralized group. The motivation for taking up this topic was the possibility of improving the accuracy of the movement of a group of robots by including dynamic time synchronization in the control algorithm. The aim of this work was to develop a two-layer synchronous motion control system for a decentralized group of mobile robots. The system consists of a master layer and a sublayer. The sublayer of the control system performs the task of tracking the reference trajectory using a single robot with a kinematic and dynamic controller. In this layer, the input and output signals are linear and angular velocity. The master layer realizes the maintenance of the desired group formation and synchronization of robots during movement. Consensus tracking and virtual structure algorithms were used to implement this level of control. To verify the correctness of operation and evaluate the quality of control for the proposed proprietary approach, simulation studies were conducted in the MATLAB/Simulink environment, followed by laboratory tests using real robots under ROS. The developed system can successfully find application in transportation and logistics tasks in both civilian and military areas.

## 1. Introduction

Currently, mobile robots are at the center of interest not only for fans of technological innovations but also for most research units dealing with topics related to autonomous systems. The design of mobile robots and their control and communication systems are being analyzed. The rapid development of computer technology allows mobile robotics to use increasingly better control, autonomy, and sensor algorithms, which result in the possibility of new and better functionality. Expanding the functionality of the robot by equipping it with many sensors requires the control system to process a very large amount of information in a very short time. Fast data analysis results in the correct operation of the control algorithm in the case of a dynamic environmental situation (the need to stop or avoid an obstacle).

Observing the development of mobile robotics in the context of Industry 4.0, it can be seen that engineers are not only focused on designing and building robots dedicated to specific tasks but also on integrating robots into groups. A robot group is a set of robots with the same or different functional and driving properties, which can cooperate with each other and exchange information to perform a task together. For certain types of tasks, such as land exploration, land demining, or large-scale transportation and assembly, the use of a group of robots can reduce the time required, minimize the risk of loss of human health or life, and increase the chances of successful task achievement.

The need to develop autonomous systems for the above-mentioned tasks is especially seen in Ukraine, where a military conflict is underway. The need for logistical support of units on the front lines is also important. According to experts, in the near future, there will be a need to develop autonomous systems precisely for these tasks, which was the motivation for this project.

The difficulties of group control lie in the realization of the interaction of robots in a common environment, resulting in the achievement of a common goal [1,2]. Engineering challenges accompanying the construction of such a system concern the development of solutions related to mutual communication, navigation, and synchronization of robots in autonomous control [3]. A very important problem in controlling a decentralized group of robots is the delay of data transmission. High delay of control data can cause inaccuracies in the trajectory of the movement of a group of robots, the behavior of which is necessary for the correct execution of the given task. The exclusion of errors caused by time drift is an integral part of the construction of the control algorithm of a decentralized group and has a significant impact on the evaluation of the quality of task execution by the group.

This work proposes an author’s system for synchronous motion control of a decentralized group of mobile robots, which are increasingly being used in the scientific community and industry because they can perform tasks faster and more efficiently than single-agent systems, as evidenced by the work of [4,5]. The solution is presented for a group of three differentially driven mobile robots. In this author’s approach, a two-layer control system, consisting of a master layer and a sublayer, is proposed. The sublayer of the control system performs the task of tracking a reference trajectory by a single robot, which was realized using a kinematic and dynamic controller, where the input and output signals are linear and angular velocity. The master layer realizes the maintenance of the set group formation and synchronization of robots during movement. The assumptions of consensus tracking and virtual structure algorithms were used to implement this level of control. To verify the correctness of operation and evaluate the quality of control for the proposed author’s approach, simulation studies were carried out in the MATLAB/Simulink environment, followed by laboratory tests using real robots with ROS. During the tests, the robustness of the control algorithm to time delays in control signals and interference from malfunctioning robots in the group was tested. The results obtained confirmed the superiority of this solution over others described.

In Section two of this work, the author presented related works. Section three presents the selected research object for which the control system was developed, as well as the theoretical model and the structure of the author’s control system. Section four contains the results of the simulation and experimental tests carried out. In Section five, the author summarizes the work carried out.

## 2. Related Works

The control of a robot formation is closely related to the type of task being performed. There are two structures that describe the methodology of controlling a group of robots: a central (hierarchical) structure and a decentralized (distributed) structure. The architecture of control systems is also linked to the adopted solution for motion or task planning. In patrol, combat, and inspection tasks, it is allowed to change the position of robots in an array while maintaining it. This is achieved using control algorithms based on tracking the position of the leader [6,7,8,9]. The idea behind this algorithm is to minimize the distance and angle of deviation from the point associated with the leader robot in successive iterative steps. Another example of the use of a follow-the-leader strategy is presented in paper [10], where an optimized formation control algorithm is presented for unmanned surface vehicles (USVs) with collision avoidance. In this work, the leader–follower algorithm is equipped with neural networks with radial basis functions (NNs) by which system uncertainties such as damping and unmodeled dynamics are approximated. The implemented neural networks are learned using the PR algorithm, which ensures optimal control of the formation. The advantage of controlling the leader-tracking method is the ease of controlling multiple robots in the desired formation. The method is ideal for creating a mathematical description of the formation. The disadvantage is the difficulty of accurately maintaining preset distances between robots.

For tasks other than exploration, where high-precision positioning of robots in a group is required (e.g., large-scale transportation, assembly, or cooperation with industrial robots), the formation must maintain a defined geometric array without the possibility of changing the order of the robots. Such tasks are implemented using the virtual structure algorithm [11,12,13]. In this method, the robots always maintain their positions relative to the reference system, which moves in space. Thus, the movement of a group of robots in space (in the global coordinate system) is realized without changing their mutual position and position relative to a common reference system. Considering a group of multiple mobile robots, the solution to the problem of moving in a formation must simultaneously achieve two goals: progress in a given direction and maintaining geometric compliance with the imposed virtual structure (moving coordinate system). However, if errors are imposed on the movement of one or more robots or tracking performance is lost, the robot will not be able to match the position perfectly with respect to the virtual structure, and the shape of the formation will be disturbed.

Another method for controlling a group of robots uses an approach based on graph theory to design a closed-loop control system with a feedback loop and model the interconnection of robots in a group. Based on [14], a graph is a set of vertices (a.k.a. nodes) and edges connecting pairs of vertices. In a simplified way, it can be assumed that a graph is a geometric figure. If it is assumed that there is no difference between two vertices associated with a given edge, then the graph is referred to as undirected. If an edge is assumed to be directed from one vertex to another, then communication between two robots in a group can only be unidirectional.

Analyzing the literature in terms of the application of the graph theory algorithm, this method is widely developed and often applied to distributed control. In the paper [15], the graph theory method was applied to the decentralized control of a group of nonholonomic wheeled robots based on their nonlinear models. In the presented solution, the robots form the required geometric pattern, the geometric center of which moves along a given reference trajectory, and the information about it is available only to some robots in the group. In turn, Ref. [16] addresses the problem of controlling the formation of nonholonomic robots in finite time. In the presented solution, a unified error system was introduced for the distributed group, and then a control law was formulated for the task of trajectory tracking by each robot. The advantage of this method is the ease of representation of any formation by means of a graph and, thus, the simplicity of mathematical description. However, this approach requires the exchange of a very large amount of information between robots, which is limited by the properties of the communication equipment.

The last control strategy for a decentralized group of robots presented in this work is the consensus algorithm. According to the consensus strategy, one way to control the group formation is to optimize the velocity profile for individual robots to maintain a preset group formation while executing a benchmark trajectory. Such a solution was presented in the paper [17], where the authors modeled the group’s formation maintenance as velocity-dependent and then reduced the motion coordination problem to a problem of optimizing the velocities of individual robots and used the linear interactive and general optimizer (Lingo) method to minimize the objective function. Another example is the work of [18], which presents a control system for a group of robots moving in an unknown environment with obstacles. The work uses a two-level control system architecture: local control for each robot and global control for maintaining the formation of the group. A decentralized local-constant-path planner was used to control and plan the trajectory for each robot. On the other hand, at the second level of control, the coordination of the movement of individual robots to form a goal formation, as well as the constraints on individual robots and their interrelationships, were presented using a ‘Constraint tree’ diagram. Then, a control method called dynamic priority strategy was implemented.

A common feature of the aforementioned [17,18] methods is the implementation of kinematic controllers to achieve and maintain a preset formation based on mutual velocity tracking of individual robots of a group. Since, in reality, the dynamics of a single robot, as well as the relationships in the group, can be affected by many factors that generate disturbances, the assumption of perfect velocity tracking does not work in practice, especially in the implementation of tasks that require precise positioning, i.e., large-volume transport. Therefore, in this case, it is necessary to implement a group control strategy that considers the kinematics and dynamics of the robots. However, in practice, perfect knowledge of the dynamic model of the mobile robot is not achievable, and it is very difficult to obtain accurate values of the model parameters [19]. Consensus algorithms are used to solve the above problem. One way to solve the problem of controlling a group of robots with unknown dynamics is presented in [19], where the transformation of formation control to a consensus problem is described. Then, kinematic controllers based on distributed consensus were developed, whose control consists of asymptotically converging the robots into a desired geometric pattern and controlling the virtual center of the group so that it moves along a designated trajectory. The algorithm assumes local information exchange between robots, where only neighboring robots have information about the state they are currently in. The described solution was implemented using a model of robot dynamics, where the output signals are torques. Maintenance of group formation is implemented using online identification of parameters of the dynamics model using neural networks. The use of a two-layer control architecture for a group of robots is a good and common solution, as also shown in [20], which describes a formation control method for multiple surface vehicles (SVs). The control algorithm consists of a high-level displacement-based formation controller and a low-level optimal control strategy based on reinforcement learning (RL) for individual agents. A modified gradient method was used as the high-level control law. As a low-level control law, the RL algorithm was used to transform a time-varying agent system into an equivalent autonomous system.

Another example of reducing the task of formation control to a consensus problem is the work of [21], which proposes a decentralized, time-varying, and continuous group controller, and [22], which presents adaptive trajectory tracking algorithms for the consensus problem. In addition, special attention was paid to the topology of communication between robots.

The aforementioned works present an asymptotic control approach. For the realization of precise tracking of the reference trajectory, finite-time control is a more effective approach. These methods are more extensively described in the [23,24]. They are characterized by short control times and good robustness to uncertainties and disturbances.

The approach of implementing the task of trajectory tracking by a group of robots using a consensus algorithm forces the processing of a very large amount of information about the state of the robots in the group. As the number of robots in the group increases and the signal transmission parameters vary, the performance of the controlled system tends to decrease and even makes the system unstable. Therefore, the study of time delays in control systems for groups of robots using the consensus algorithm is becoming an important research direction in the field of control, which has been addressed in [25,26,27] and in this work.

## 3. Materials and Methods

**Figure 1:**
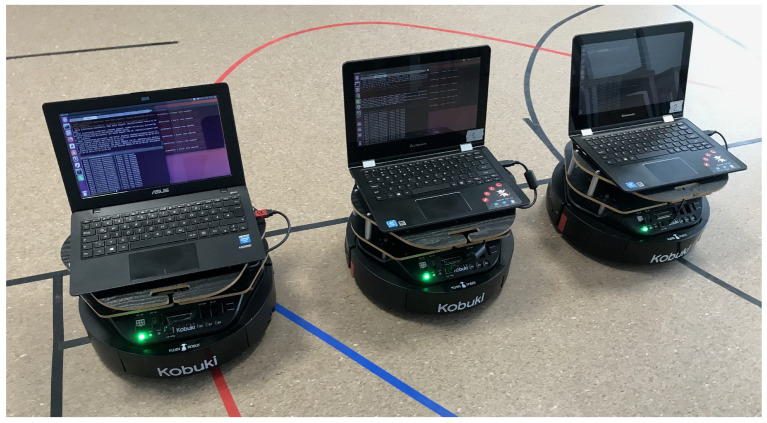
Hardware structure of the autonomous vehicle.

The considerations presented in this paper include a group of TURTLEBOT 2 laboratory robots equipped with the robot operating system (ROS Kinetic and Ubuntu 16.04). The TURTLEBOT 2 robot is a two-wheeled, differential-driven mobile robot with two support wheels. An additional advantage in favor of using this robot is its chassis system, which is a base for expansion to the chassis systems of AGV robots used in the industry. Three TURTLEBOT 2 robots with a modified design were used to build a group with a distributed structure, as shown in Figure 1.

Due to the chosen object of research, it was necessary to develop a control system in the robot operating system (ROS), which TURTLEBOT 2 robots are equipped with. ROS is a software platform for developing software for robots, which can be regarded as a system with a client/server structure. The platform is equipped with its own mechanism for building code into executable programs. This ensures that the correct order of attaching libraries, dependencies with other modules, and running tools is the same for each module. This saves time when looking for errors both during compilation and program execution.

For the robots described above, a two-layer control system consisting of a master layer and a sub-layer was built. The sub-layer of the control system performs the task of tracking a reference trajectory by a single robot, which was implemented using a kinematic and dynamic controller, where the input and output signals are linear and angular velocity. The master layer realizes the maintenance of the formation of a group of sets and the synchronization of robots during movement. The assumptions of consensus tracking and virtual structure algorithms were used to implement this level of control. Next, simulation studies were conducted in the MATLAB R2023a/Simulink environment, followed by laboratory tests, which consisted of testing the accuracy of the group’s movement along a given trajectory first without considering the developed control method and then with considering the developed method. During the simulation and laboratory tests, the robustness of the control algorithm to time delays in control signals and disturbances caused by malfunctioning robots in a group was tested, as well as the ability of the developed solution to minimize distance errors between robots in a group.

### 3.1. The Kinematics and Dynamics Model of Robots

The theoretical model of the TURTLEBOT 2 robot is divided into two parts: a kinematic model and a dynamic model. The derivation of each model is presented in [28,29].

**Figure 2:**
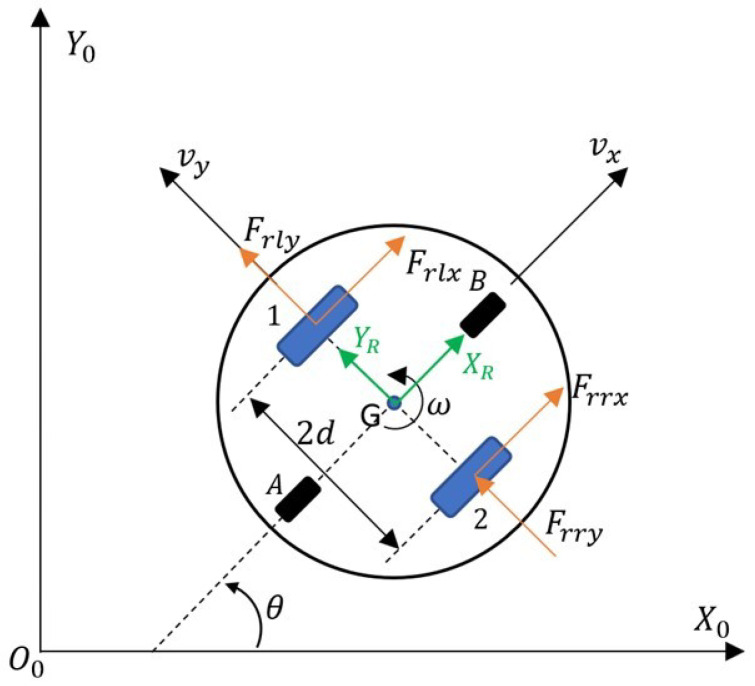
Kinematic and dynamic structure of the TURTLEBOT 2 robot.

The diagram shown in Figure 2 was used to determine the kinematic and dynamic model of the TURTLEBOT 2 robot.

The $O_{0}X_{0}Y_{0}$ coordinate system is a global, stationary reference system. The *x* and *y* coordinates define the positions, the angle θ, and the orientation of the robot in the global coordinate system. Associated with the base of the robot is a moving coordinate system $GX_{R}Y_{R}$ with an origin at the geometric center of the robot *G*. The point *G* shown in the TURTLEBOT 2 robot schematic is also the robot’s center of rotation and the tracking point for the desired trajectory. Vector $v_{x}$ determines the linear forward velocity, $v_{y}$ lateral linear velocity, while ω determines the angular velocity of the robot. Numbers 1 and 2 denote the drive wheels, while letters A and B denote the support wheels. The kinematics of the TURTLEBOT 2 mobile robot are described by Equation (1) in a form in which the control signals are the linear and angular speed of the robot [28].

(1) $$\left[\begin{array}{c} \dot{x}\\ \dot{y}\\ \dot{\theta } \end{array}\right]=\left[\begin{array}{cc} cos\theta & 0\\ sin\theta & 0\\ 0 & 1 \end{array}\right]\left[\begin{array}{c} v_{x}\\ \omega \end{array}\right].$$

In Figure 2, the robot’s driving forces are labeled as $F_{rrx}$, $F_{rry}$, the longitudinal and lateral force of the right wheel and $F_{rlx}$, $F_{rly}$, and the longitudinal and lateral force of the left wheel. The dynamics model of the robot in discussion is presented by a system of Equations (2) [28,29]:

(2) $$\left[\begin{array}{c} {\dot{v}}_{x}\\ \dot{\omega } \end{array}\right]=\left[\begin{array}{c} -\frac{\sigma_{3}}{\sigma_{1}}v_{x}\\ -\frac{\sigma_{4}}{\sigma_{2}}\omega \end{array}\right]+\left[\begin{array}{cc} \frac{1}{\sigma_{1}} & 0\\ 0 & \frac{1}{\sigma_{2}} \end{array}\right]\left[\begin{array}{c} v_{xd}\\ \omega_{d} \end{array}\right]\;.$$

where *m* is the mass of the robot, *r* is the radius of the drive wheels, $k_{b}$ is the electrical constant of the motors, $k_{a}$ is the mechanical constant of the motors, $R_{a}$ is the resistance of the motor windings, $I_{e}$ is the moment of inertia of the wheel with respect to the axis of rotation, $B_{e}$ is the coefficient of viscous friction reduced to the motor shaft, and $k_{PT}$, $k_{DT}$, $k_{PR}$, and $k_{DR}$ are the gains of the built-in PD controller.

The dynamic model of the robot contains unknown parameters, $\sigma_{1}$, …, $\sigma_{4}$, which must be identified. The process of identifying these parameters is presented in [28]. A group of cooperating robots can be described by a group state vector H, which is a composite of the states of individual robots [14,30]. When considering a group control system with a distributed structure, the equation of the state of each robot is a function of its state vector $h_{i}\left(t\right)$ and the input–control vector $u_{i}\left(t\right)$. The state vector of the distributed group can be defined as follows:

(3) $$H\left(t\right)={\left[h_{1}\left(t\right),h_{2}\left(t\right),\ldots ,h_{n}\left(t\right)\right]}^{T},$$

and the equation of state of each robot can be taken as follows:

(4) $${\dot{h}}_{i}\left(t\right)=f_{i}\left(h_{i}\left(t\right),u_{i}\left(t\right)\right),$$

where the control vector $u_{i}$ takes the form of the following:

(5) $$u_{i}\left(t\right)=k_{i}\left(h_{i}\left(t\right),e_{i}\left(t\right)\right),$$

and the error vector $e_{i}$ is as follows:

(6) $$e_{i}\left(t\right)=h_{i0}\left(t\right)-h_{i}\left(t\right)\;,$$

where the equation of state of a decentralized group of robots can be formulated as follows:

(7) $$\dot{H}\left(t\right)=F\left(H\left(t\right)\right),\;\mathrm{where}\;H\left(t\right)={\left[f_{1}\left(t\right),f_{2}\left(t\right),\ldots ,f_{n}\left(t\right)\right]}^{T}.$$

Considering a group of TURTLEBOT 2 robots, we can define a vector of configuration coordinates (state) of each robot in the form of the following:

(8) $$h_{i}\left(t_{i}\right)=\left[x_{i}\left(t_{i}\right),y_{i}\left(t_{i}\right),\theta_{i}\left(t_{i}\right),v_{xi}\left(t_{i}\right),v_{yi}\left(t_{i}\right),\omega_{i}\left(t_{i}\right)\right].$$

We can assume a vector of controls with synchronization (time delays) in the form the following:

(9) $$u_{i}\left(t_{i}+\Delta t_{i}\right)=\left[v_{x_{id}}\left(t_{i}+\Delta t_{i}\right),v_{x_{id}}\left(t_{i}+\Delta t_{i}\right),\omega_{id}\left(t_{i}+\Delta t_{i}\right)\right],$$

where the individual inputs correspond to linear velocity $v_{i}$ and angular velocity $\omega_{i}$. As a result, one can formulate the equation of state considering the time synchronization for each robot as follows:

(10) $$\begin{array}{c} {\dot{h}}_{i}\left(t_{i}\right)=f_{i}\left(h_{i}\left(t_{i}\right),u_{i}\left(t_{i}+\Delta t_{i}\right)\right)\to \left[\begin{array}{c} {\dot{x}}_{i}\left(t_{i}\right)\\ {\dot{y}}_{i}\left(t_{i}\right)\\ {\dot{\theta }}_{i}\left(t_{i}\right)\\ {\dot{v}}_{xi}\left(t_{i}\right)\\ {\dot{v}}_{yi}\left(t_{i}\right)\\ {\dot{\omega }}_{i}\left(t_{i}\right) \end{array}\right]=\left[\begin{array}{c} v_{xi}\left(t_{i}+\Delta t_{i}\right)cos\theta_{i}\left(t_{i}+\Delta t_{i}\right)\\ v_{yi}\left(t_{i}+\Delta t_{i}\right)sin\theta_{i}\left(t_{i}+\Delta t_{i}\right)\\ \omega_{i}\left(t_{i}+\Delta t_{i}\right)\\ -\frac{\sigma_{3_{i}}}{\sigma_{1_{i}}}v_{xi}\left(t_{i}+\Delta t_{i}\right)\\ -\frac{\sigma_{3_{i}}}{\sigma_{1_{i}}}v_{yi}\left(t_{i}+\Delta t_{i}\right)\\ -\frac{\sigma_{4_{i}}}{\sigma_{2_{i}}}\omega_{i}\left(t_{i}+\Delta t_{i}\right) \end{array}\right]+\\ +\left[\begin{array}{ccc} 0 & 0 & 0\\ 0 & 0 & 0\\ 0 & 0 & 0\\ \frac{1}{\sigma_{1_{i}}} & 0 & 0\\ 0 & \frac{1}{\sigma_{1_{i}}} & 0\\ 0 & 0 & \frac{1}{\sigma_{2_{i}}} \end{array}\right]\left[\begin{array}{c} v_{x_{id}}\left(t_{i}+\Delta t_{i}\right)\\ v_{y_{id}}\left(t_{i}+\Delta t_{i}\right)\\ \omega_{i_{d}}\left(t_{i}+\Delta t_{i}\right) \end{array}\right], \end{array}$$

and the equation of state of the dispersed group is as follows:

(11) $$\dot{H}\left(t\right)=F\left(H\left(t\right)\right),\;\mathrm{where}\;H\left(t\right)={\left[f_{1}\left(t_{1}+\Delta t_{1}\right),f_{2}\left(t_{2}+\Delta t_{2}\right),\ldots ,f_{n}\left(t_{n}+\Delta t_{n}\right)\right]}^{T}.$$

The equations presented here model the continuous behavior of each robot, and consequently, the continuous behavior of the group when performing a group task [14,30,31,32,33].

### 3.2. Synchronous Control Algorithm for a Decentralized Group of Robots

**Figure 3:**
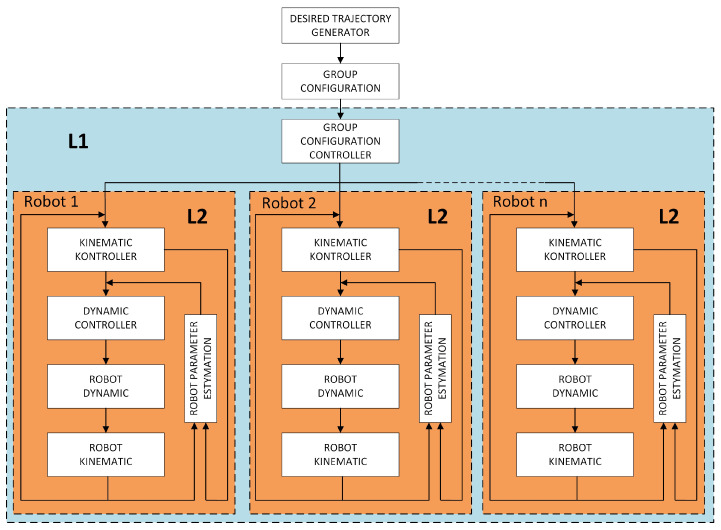
Structure of a two-layer control system for a decentralized group of mobile robots.

For the case of the decentralized structure group under consideration, a two-layer control algorithm has been proposed, as shown in Figure 3:

The L1 layer of group master control is responsible for maintaining the required group formation. The L2 layer of slave control is responsible for controlling individual robots performing the task of trajectory tracking. The algorithm consists of several software modules performing specific functions, which are described in the following subsections.

In the block DESIRED TRAJECTORY GENERATOR, the reference trajectory of the virtual structure (moving coordinate system against which the formation is built) realizing the given (reference) trajectory for the group ($x_{d}\left(t\right)$,$y_{d}\left(t\right)$) and its orientation $\theta_{d}\left(t\right)$ relative to the global coordinate system is defined. Then, if the robot moves along the given trajectory without interference and without initial errors, the reference control signals $u_{1}\left(t\right)$ and $u_{2}\left(t\right)$, are determined, which are the reference linear and angular velocities, respectively, in the trajectory tracking task. The reference orientation angle $\theta_{d}\left(t\right)$ at point ($x_{d}\left(t\right)$,$y_{d}\left(t\right)$) is determined by the following relationship:

(12) $$\theta_{d}\left(t\right)=arctan2\left({\dot{x}}_{d}\left(t\right),{\dot{y}}_{d}\left(t\right)\right)+k\pi ,$$

where k=0,1 corresponds to the direction of rotation (for k=0 rotation to the left, for k=1 rotation to the right). The linear velocity of the group is determined by the following relationship:

(13) $$u_{1}\left(t\right)=v_{d}\left(t\right)=\pm \sqrt{{\dot{x}}_{d}^{2}\left(t\right)+{\dot{y}}_{d}^{2}\left(t\right)},$$

where the sign depends on the direction of movement (+ declares forward movement, − declares backward movement). Differentiating Equation (12) in the time domain, a relation for determining the reference angular velocity of the group was obtained:

(14) $$u_{2}\left(t\right)=\omega_{d}\left(t\right)=\frac{{\dot{x}}_{d}\left(t\right){\ddot{y}}_{d}\left(t\right)-{\dot{y}}_{d}\left(t\right){\ddot{x}}_{d}\left(t\right)}{{\dot{x}}_{d}^{2}\left(t\right)+{\dot{y}}_{d}^{2}\left(t\right)}.$$

Combining the above equations, a vector of $q_{d}\left(t\right)$ pattern configuration coordinates was obtained:

(15) $$q_{d}\left(t\right)={\left[x_{d}\left(t\right),y_{d}\left(t\right),\theta_{d}\left(t\right)\right]}^{T}\;.$$

**Figure 4:**
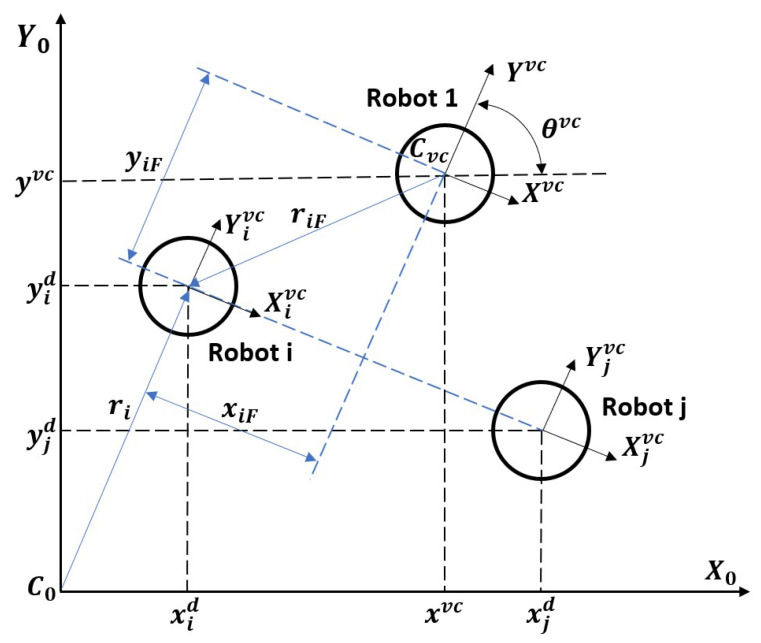
Interaction of robots in a distributed group with the assumptions of the virtual structure algorithm.

In the ROBOT GROUP CONFIGURATION block, the shape of the formation is defined, and the position of the robots included in the group is determined. The assumptions of the virtual structure algorithm [33] were used to model the formation. The diagram of the interrelations of robots in the distributed group is shown in Figure 4. The center point of the virtual structure is in the geometric center of Robot 1, whose coordinates and orientation are determined by the vector $q_{1}\left(t\right)=\left[x_{1}^{d}\left(t\right),y_{1}^{d}\left(t\right),\theta_{1}^{d}\left(t\right)\right]$. Angle $\theta_{1}^{d}\left(t\right)$ is the same as angle $\theta_{i}^{vc}\left(t\right)$, which determines the orientation of the group in the global coordinate system $C_{0}X_{0}Y_{0}$. The position of the other robots in the group is determined by rigid distances $x_{iF}$ and $y_{iF}$ from the center of the virtual structure’s coordinate system.

Robot coordinates for any configuration and number of robots relative to the coordinate system of the virtual structure are determined using the following equations:

(16) $$\left[\begin{array}{c} x_{i}^{d}\left(t\right)\\ y_{i}^{d}\left(t\right) \end{array}\right]=\left[\begin{array}{c} x_{i}^{vc}\left(t\right)\\ y_{i}^{vc}\left(t\right) \end{array}\right]+\left[\begin{array}{cc} cos(\theta_{i}^{vc}\left(t\right)) & -sin(\theta_{i}^{vc}\left(t\right))\\ sin(\theta_{i}^{vc}\left(t\right)) & cos(\theta_{i}^{vc}\left(t\right)) \end{array}\right]\left[\begin{array}{c} x_{iF}\\ y_{iF} \end{array}\right].$$

GROUP CONFIGURATION CONTROLLER (see Figure 4) was developed using the considerations presented in [34,35,36,37]. To use consensus algorithms to maintain group formation, it is assumed that the group’s model trajectory (position and orientation of the virtual coordinate system) for each robot is known. If there is a risk of a lack of information exchange between the robots due to a dynamically changing environment, limited information exchange or communication loss, the desired group formation may not be maintained. In this case, a group state estimator for each robot should be introduced in the group control algorithm [34].

Taking the actual position in the reference system $C_{0}$ of the *i*-th robot in the group as $r_{i}\left(t\right)={\left[x_{i}\left(t\right),y_{i}\left(t\right)\right]}^{T}$, the position of the geometric center of the virtual structure as $C_{vc}\left(t\right)=\left[x^{vc}\left(t\right),y^{vc}\left(t\right)\right]$, its orientation $\theta^{vc}\left(t\right)$ relative to the reference system, and the deviation of the *i*-th robot in the group with respect to the center of the virtual structure as $r_{iF}\left(t\right)=\left[x_{iF}\left(t\right),y_{iF}\left(t\right)\right]$, the reference position of the *i*-th robot included in the group can be determined as $r_{i}^{d}\left(t\right)={\left[x_{i}^{d}\left(t\right),y_{i}^{d}\left(t\right)\right]}^{T}$ using the following relation:

(17) $$\left[\begin{array}{c} x_{i}^{d}\left(t\right)\\ y_{i}^{d}\left(t\right) \end{array}\right]=\left[\begin{array}{c} x^{vc}\left(t\right)\\ y^{vc}\left(t\right) \end{array}\right]+\left[\begin{array}{cc} cos(\theta^{vc}\left(t\right)) & -sin(\theta^{vc}\left(t\right))\\ sin(\theta^{vc}\left(t\right)) & cos(\theta^{vc}\left(t\right)) \end{array}\right]\left[\begin{array}{c} x_{iF}\\ y_{iF} \end{array}\right].$$

If each robot in the group follows its reference position without error, then the group’s formation is maintained. In reality, the occurance of errors in the transmission of information between robots regarding their current state leads to disturbances in the maintenance of the formation. Therefore, the dynamics of a multi-agent system with time-dependent ties can be written based on [36] as follows:

(18) $$\xi_{i}\left(t\right)=h\left(t,\xi_{i}\right)+\sum_{j=1}^{m}g_{ij}f\left(t,\xi_{j}\left(t+\Delta t_{ij}\right),\xi_{i}\left(t+\Delta t_{ij}\right)\right),\;\;\;\;i=1,2,\ldots ,m,$$

where $\xi_{i(t)}=\left[x_{i(t)},y_{i(t)},\theta_{i(t)}\right]$ is the state of robot i at time t, $g_{ij}$ is the weight determining the communication links between robots i and j, and $\Delta t_{ij}$ is the time delay in communication between robots *i* and *j*.

Specifying the position of the robot at time t as $r_{i}\left(t\right)=\left[x_{i}\left(t\right),y_{i}\left(t\right)\right]$ and the control signal of the robot i in the group as $u_{i}\left(t\right)$, the basic control law for the consensus algorithm takes the following form (19):

(19) $$u_{i}\left(t\right)=-\sum_{j=1}^{n}g_{ij}k_{ij}f\left(r_{i}\left(t\right)-r_{j}\left(t\right)\right),\;\;\;\;i=1,2,\ldots ,n,$$

where $k_{ij}$ is the controller gain for a pair of robots ij, and $g_{ij}=1$ if there is data transmission from robot j to robot i. $g_{ij}=0$ if transmission does not occur. Consensus between robots i and j will be reached if Equation (20) is satisfied:

(20) $$\lim_{t\to \infty }\left\|\xi_{i}\left(t\right)-\xi_{j}\left(t\right)\right\|=0.$$

If all pairs of robots in a group reach consensus (satisfy Equation (20)), the entire dynamic system will reach group consensus, i.e., the model formation will be preserved [34,35,36].

**Figure 5:**
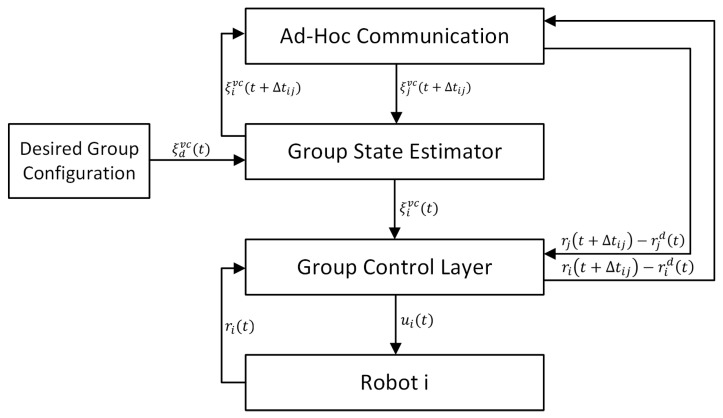
Block diagram of the decentralized group controller.

The decentralized group controller was developed as a hierarchical structure containing two layers: the group state estimator and the group robot control module, as shown in Figure 5.

The decentralized structure group controller is built with two control layers: the group state estimator and the group robot control module, as shown in Figure 5. Denoting the group state (position and orientation of the virtual structure coordinate system) as $\xi^{vc}\left(t\right)={\left[x^{vc}\left(t\right),y^{vc}\left(t\right),\theta^{vc}\left(t\right)\right]}^{T}$, the group reference state as $\xi_{d}^{vc}\left(t\right)={\left[x^{vc}\left(t\right),y^{vc}\left(t\right),\theta^{vc}\left(t\right)\right]}^{T}$, and the actual state of the *i*-th robot as $\xi_{i}^{vc}\left(t\right)={\left[x_{i}^{vc}\left(t\right),y_{i}^{vc}\left(t\right),\theta_{i}^{vc}\left(t\right)\right]}^{T}$, a control law, was formulated for the group state estimator, which takes into account time delays in communication between robots in the form of Equation (21), based on which the state of the coordinate system of the virtual structure for each robot is estimated as follows:

(21) $${\dot{\xi }}_{i}^{vc}\left(t\right)=\frac{{\dot{\xi }}_{d}^{vc}\left(t\right)-\gamma \left(\xi_{i}^{vc}\left(t\right)-\xi_{d}^{vc}\left(t\right)\right)+\sum_{j=1}^{n}g_{ij}^{vc}\left[{\dot{\xi }}_{j}^{vc}\left(t\right)-\gamma \left(\xi_{i}^{vc}\left(t+\Delta t_{ij}\right)-\xi_{j}^{vc}\left(t+\Delta t_{ij}\right)\right)\right]}{1+\sum_{j=1}^{n}g_{ij}^{vc}},$$

where $g_{ij}^{vc}=1$ if robot i receives information from the group state estimator about the state of robot *j*, $g_{ij}^{vc}=0$ if robot i does not receive information about the status of robot *j*, and γ>0 is the gain coefficient. In addition, it was assumed that each robot receives information about the state of the virtual coordinate system and the value of the time delay in communication between robots; therefore, the group state estimator should provide $\xi_{i}^{vc}\left(t\right)\to \xi_{d}^{vc}\left(t\right)$.

As a control layer for the robots in the group based on [36], the control law of the Extended Consensus Algorithm (22) was used, which takes into account time delays in communication between robots. Equation (22) allows us to determine the control signals $u_{i}\left(t\right)$ so that the model geometric formation is maintained during movement:

(22) $$u_{i}\left(t\right)={\dot{r}}_{i}^{d}\left(t\right)-\alpha_{i}\left(r_{i}\left(t\right)-r_{i}^{d}\left(t\right)\right)-\sum_{j=1}^{n}g_{ij}k_{ij}\left[\left(r_{i}\left(t+\Delta t_{ij}\right)-r_{i}^{d}\left(t\right)\right)-\left(r_{j}\left(t+\Delta t_{ij}\right)-r_{j}^{d}\left(t\right)\right)\right].$$

Implementation of the task of tracking a reference trajectory by a robot is always associated with the appearance of errors, i.e., the difference between the reference position and the actual position. For this purpose, a proportional KINEMATIC CONTROLLER (Figure 3) with a feedback loop, extensively described in [28], was proposed, whose task is to obtain control signals directing the robot to the set trajectory.

The proposed control law makes it possible to determine two control signals: linear velocity and angular velocity based on the transformation of the robot’s position and orientation errors and reference velocities. The kinematic controller equation takes the following form:

(23) $$\left[\begin{array}{c} u_{kv}\left(t\right)\\ u_{k\omega }\left(t\right) \end{array}\right]=\left[\begin{array}{c} v_{d}\left(t\right)cos\theta_{d}\left(t\right)+k_{1}e_{1}\left(t\right)\\ \omega_{d}\left(t\right)+v_{d}\left(t\right)\left(k_{2}e_{2}\left(t\right)+k_{3}sine_{3}\left(t\right)\right)] \end{array}\right],$$

where $u_{kv}\left(t\right)$ and $u_{k\omega (t)}$ are the control signals, $v_{d}\left(t\right)$ and $\omega_{d}\left(t\right)$ are the reference linear and angular velocities, and $k_{1}$, $k_{2}$, and $k_{3}$ are the controller gains. The trajectory tracking errors, $e_{1}\left(t\right)$, $e_{2}\left(t\right)$, and $e_{3}\left(t\right)$, can be determined using the following formula:

(24) $$\left[\begin{array}{c} e_{1}\left(t\right)\\ e_{2}\left(t\right)\\ e_{3}\left(t\right) \end{array}\right]=\left[\begin{array}{ccc} cos\theta (t) & sin\theta (t) & 0\\ -sin\theta (t) & cos\theta (t) & 0\\ 0 & 0 & 1] \end{array}\right]\left[\begin{array}{c} x_{r}\left(t\right)-x_{d}\left(t\right)\\ y_{r}\left(t\right)-y_{d}\left(t\right)\\ \theta_{r}\left(t\right)-\theta_{d}\left(t\right)] \end{array}\right],$$

where $x_{r}\left(t\right)$, $y_{r}\left(t\right)$ is the actual position of the robot, and $\theta_{r}\left(t\right)$ is the actual orientation of the robot.

The determination of the controller’s gains was carried out by comparing the actual and desired elements of the characteristic polynomial as in [38]. The solutions to the system of equations comparing the characteristic elements of the polynomial are the gains described by the following relations:

(25) $$\begin{array}{c} k_{1}=k_{3}=2\epsilon \omega_{n}\left(t\right)\\ k_{2}=g\left|v_{d}\left(t\right)\right| \end{array},$$

where $\omega_{n}\left(t\right)$ is characteristic frequency of the system oscillation, ϵ is the oscillation damping coefficient, and *g* is the additional control gain of the controller provided by the following formula:

(26) $$\omega_{n}\left(t\right)=\sqrt{\omega_{d}^{2}\left(t\right)+gv_{d}^{2}\left(t\right)},\;\;\;\;\epsilon \in \left(0,1\right),\;\;\;\;g>0.$$

The assumption for developing the DYNAMIC CONTROLLER (Figure 3) was to treat the control signals from the kinematic controller (Equation (23)) (linear and angular velocities) as reference signals. The process of developing the applied dynamic controller is extensively described in [28]. The formulation of the control law is described by the following equation:

(27) $$\left[\begin{array}{c} u_{dv}\left(t\right)\\ u_{d\omega }\left(t\right) \end{array}\right]=\left[\begin{array}{cc} \sigma_{1} & 0\\ 0 & \sigma_{2}] \end{array}\right]\left[\begin{array}{c} \vartheta_{1}\left(t\right)\\ \vartheta_{2}\left(t\right) \end{array}\right]+\left[\begin{array}{cccc} 0 & 0 & v_{x}\left(t\right) & 0\\ 0 & 0 & 0 & \omega (t) \end{array}\right]{\left[\begin{array}{cccc} \sigma_{1} & \sigma_{2} & \sigma_{3} & \sigma_{4} \end{array}\right]}^{T},$$

where

(28) $$\vartheta_{1}\left(t\right)={\dot{u}}_{kv}\left(t\right)+k_{v}\left(u_{kv}\left(t\right)-v_{x}\left(t\right)\right),\;\;\;\;k_{v}>0\;\vartheta_{2}\left(t\right)={\dot{u}}_{k\omega }\left(t\right)+k_{\omega }\left(u_{k\omega }\left(t\right)-\omega \left(t\right)\right),\;\;\;\;k_{\omega }>0.$$

To improve the quality of trajectory tracking using the dynamic controller (Equation (27)), adaptation of parameters $\sigma_{1}$,…,$\sigma_{4}$ during movement was proposed, as presented in [28].

## 4. Experiments and Evaluation

The correctness of the control algorithm of the decentralized group of robots was checked first by simulation and then by laboratory tests using real TURTLEBOT 2 robots. The studies were performed to check the robustness of the control algorithm to time delays in control signals and interference from malfunctions of the robots in the group.

The robustness of the control algorithm to time delays and disturbances resulting from malfunctions of the robots in the group was tested in a study of synchronous motion along a circular trajectory. In this case, the task for the group was to traverse a path equal to 3/4 of a circle with a radius of 2 m, with a declared linear velocity $v_{d}=0.1$ m/s. In the 60th second of movement, a disturbance was introduced in the form of a sudden reduction in the velocities of Robot 2 simulating, for example, resistance or damage to the drive train or a sudden fall in battery charge. In the presented test results, a 5% reduction in Robot 2’s velocity was taken into account. The test was first performed without considering the W2 layer, the group configuration controller. Then, the same test was repeated for the complete configuration of the control algorithm, including all the elements shown in Figure 5.

The position of the geometric center of the virtual structure $C_{vc}$ was fixed at the geometric center of Robot 1. The orientation $\theta^{vc}$ of the coordinate system of the virtual structure was also fixed according to the orientation of Robot 1.

The results of the study are presented in the form of graphs:

- Comparison of the reference trajectory with the simulation trajectory and the real trajectory;
- The distance error between the robots determined from the relationship:
  (29) $$e_{dij}=L_{ij}+\sqrt{{\left(x_{i}-x_{j}\right)}^{2}+{\left(y_{i}-y_{j}\right)}^{2}};$$
- Simulation and real velocities.

### 4.1. Simulation Study of Synchronous Movement of a Group along a Circular Trajectory

Simulation studies were conducted in the MATLAB/Simulink environment. The group formation was defined as a straight line. The robots were spaced 0.5 m apart, and their initial positions were described by configuration coordinate vectors in the following form:

- Robot 1: $h_{1}\left(t_{0}\right)=\left[0,0,0,0,0\right]$;
- Robot 2: $h_{2}\left(t_{0}\right)=\left[0,0.5,0,0,0\right]$;
- Robot 3: $h_{3}\left(t_{0}\right)=\left[0,-0.5,0,0,0\right]$.

**Figure 6:**
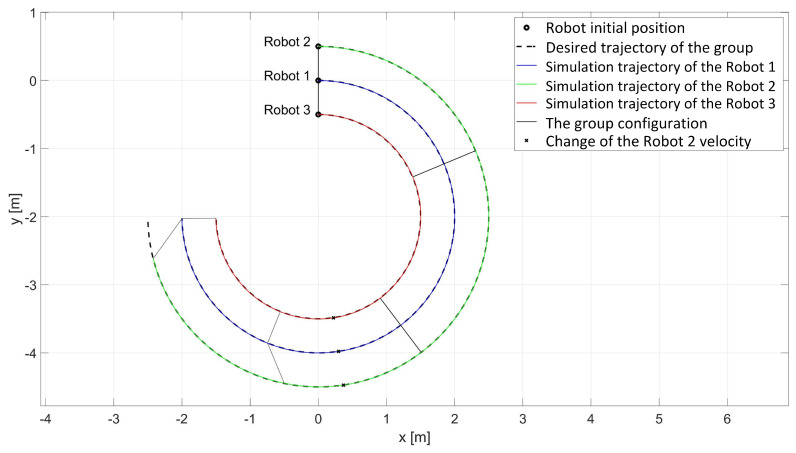
Simulation trajectories of robots in the study of synchronous motion without considering the group configuration controller.

**Figure 7:**
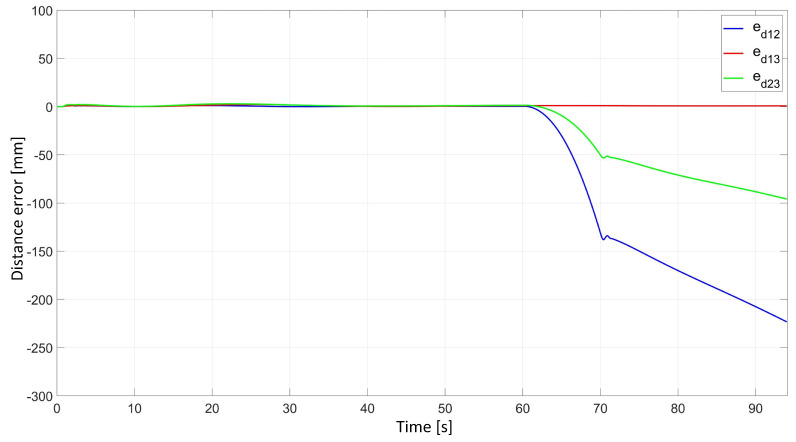
Distance errors between robots in synchronous motion study without considering the group configuration controller.

**Figure 8:**
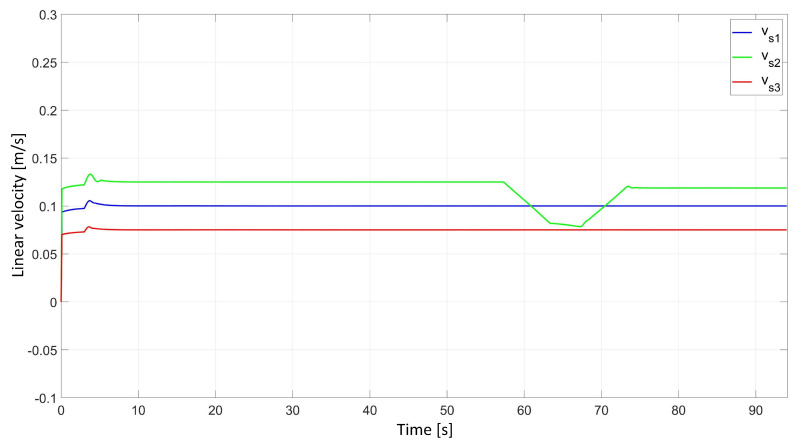
Linear velocities of robots determined by simulation in the study of synchronous motion without considering the group configuration controller.

In the simulation study of synchronous movement of the group along the given trajectory without the group configuration controller, the robots moved along the given trajectory, but because of a slight change in the velocity of Robot 2, the configuration of the group during the movement was not maintained, which can be seen in Figure 6. Analyzing the graphs of distance error (Figure 7), it can be noted that as a result of a 5% reduction in Robot 2’s velocity, large distance errors between robots appeared, which eventually reached the values $e_{d12}$ = 55.8 mm, $e_{d13}$ = 1 mm, and $e_{d23}$ = 23.9 mm, and a large tracking error of the trajectory was declared by Robot 2 with a maximum value of $eR_{2}$ = 0.58 m. Analyzing the velocity plot (Figure 8), it is noted that the robots moved at the declared velocity, but when the velocity of Robot 2 changed, the other robots did not react and continued to move at the declared velocity, deforming the established group formation.

**Figure 9:**
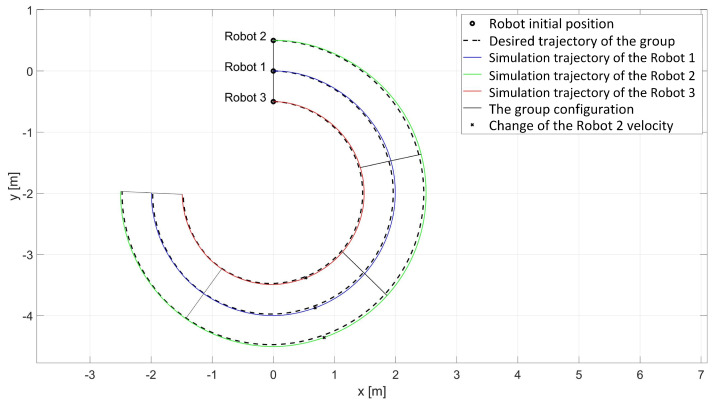
Simulation trajectories of robots in the study of synchronous motion with a group configuration controller.

**Figure 10:**
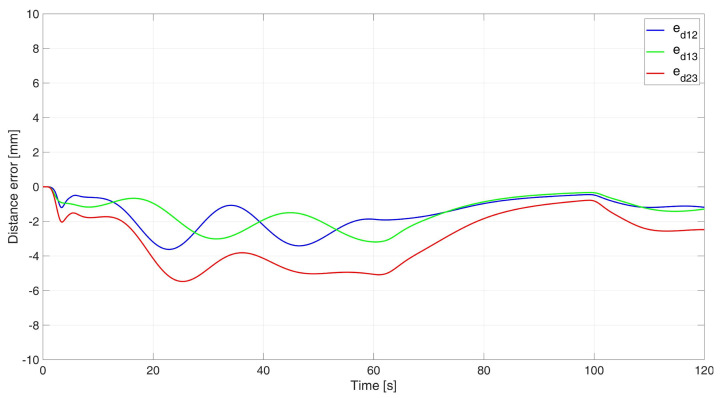
Distance errors between robots in synchronous motion study with a group configuration controller.

**Figure 11:**
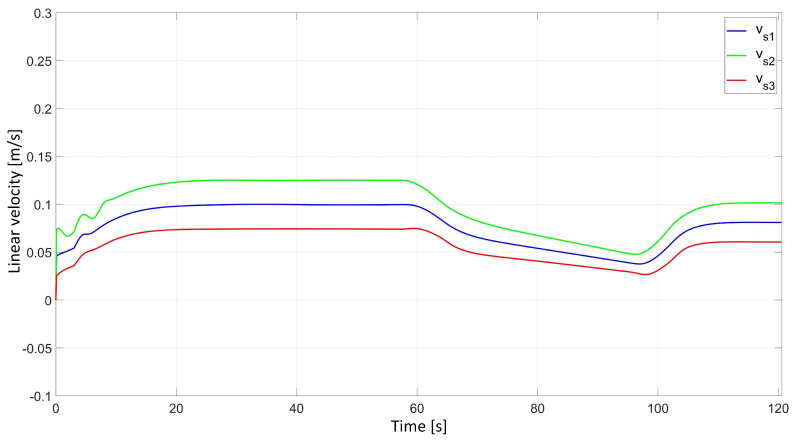
Linear velocities of robots determined by simulation in the study of synchronous motion with a group configuration controller.

In a simulation study of the synchronous movement of a group along a given trajectory with the group configuration controller, the robots moved along the given trajectory and, despite a slight change in the velocity of Robot 2, the configuration of the group during movement was maintained as can be seen in Figure 9. Analyzing the graphs of distance error (Figure 10), it can be seen that, throughout the movement, the distance error between robots was minimized and maintained, and its values were acceptable and not greater than $e_{d12}$ = 3.6 mm, $e_{d13}$ = 3 mm, and $e_{d23}$ = 5.5 mm. The trajectory tracking errors by individual robots in this study reached values no greater than $eR_{1}$ = 0.034 m, $eR_{2}$ = 0.042 m, and $eR_{3}$ = 0.035 m. Analyzing the velocity plot (Figure 11), it was noted that the robots moved at the declared velocity, and in the case of a change in the velocity of Robot 2, other robots reacted and adjusted their velocity to maintain the defined formation.

### 4.2. Laboratory Tests of Trajectory Tracking by a Group of Robots with a Decentralized Structure

During the experimental testing of the W1 layer, the results obtained from simulation studies of the synchronous start of movement of a group of robots and synchronous movement of the group along a given trajectory were verified. In the studies of the W1 layer, the configuration of the W2 layer control system shown in Figure 3 was used, which included the combination of a kinematic controller (Equation (23)) and a dynamic controller (Equation (27)) with adaptation, which showed the best control results in the studies presented in [28], and three TURTLEBOT 2 robots forming a group. To evaluate the quality of the group controller, testing was carried out in two variants. First, the study was conducted without considering the W2 layer, the consensus-based group controller. Then, the same test was repeated for the complete configuration of the control algorithm, including all elements shown in Figure 3. Communication between robots was implemented using Wi-Fi in Ad-Hoc mode, which has a decentralized structure, and devices connected to it can act as both a client and an access point. Communication between clients connected to the network is carried out in a direct way, i.e., packets are delivered to clients without the need for nodes managing communication. The adhoc_communication library of the ROS platform was used to implement programmatic communication.

**Figure 12:**
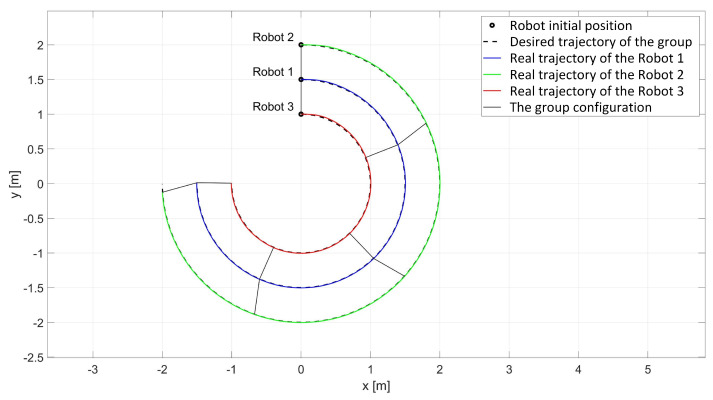
Real trajectories of robots in the study of synchronous motion without a group configuration controller.

**Figure 13:**
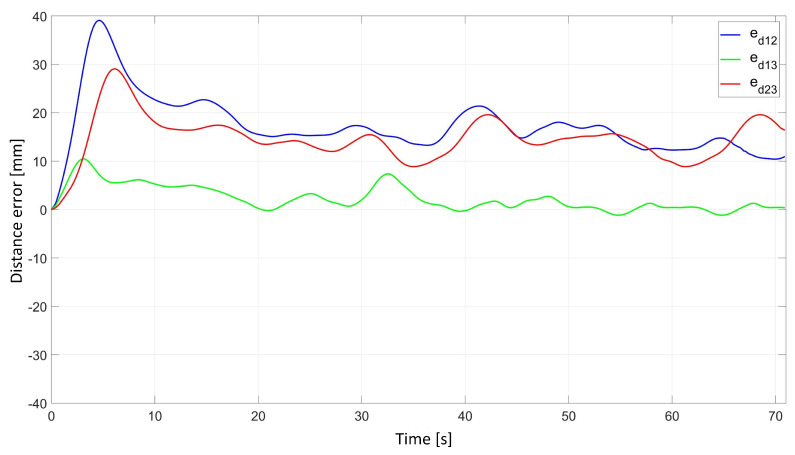
Distance errors between robots determined in the experimental study of synchronous motion without a group configuration controlle.

**Figure 14:**
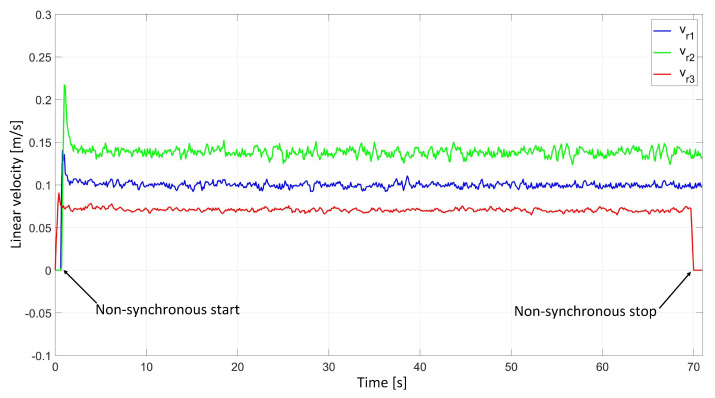
Linear velocities of robots determined in the experimental study of synchronous motion without a group configuration controller.

In the experimental study of the synchronous movement of the group along the given trajectory without the group configuration controller, the robots moved along the given trajectory, but as a result of the delay in the control signals, the configuration of the group during the movement was not maintained, which can be seen in Figure 12. Analyzing the plots of distance errors (Figure 13), it can be noted that large distance errors between the robots appeared, which reached maximum values: $e_{d12}$ = 39.6 mm, $e_{d13}$ = 10.9 mm, and $e_{d23}$ = 27.8 mm. The trajectory tracking errors of individual robots were no greater than $eR_{1}$ = 0.05 m, $eR_{2}$ = 0.06 m, and $eR_{3}$ = 0.04 m, and eventually stabilized at a value of about 0.01 m. Analyzing the velocities plot (Figure 14), it was noted that the robots moved at the declared velocity, but the start of Robot 1 and Robot 2 was delayed with respect to Robot 3.

**Figure 15:**
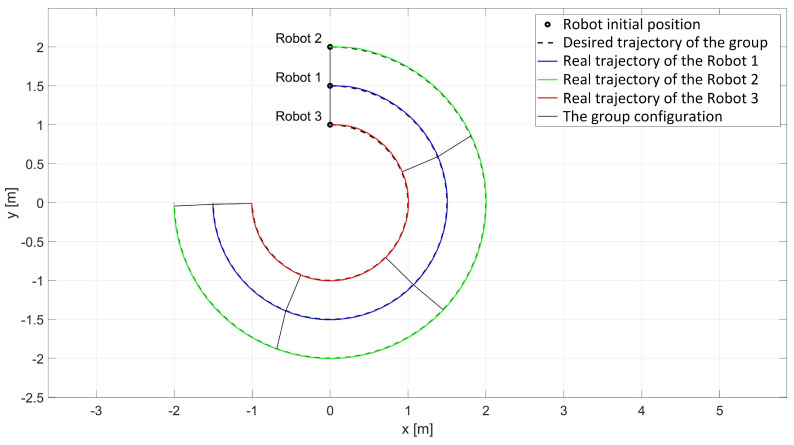
Real trajectories of robots in the study of synchronous motion with a group configuration controller.

**Figure 16:**
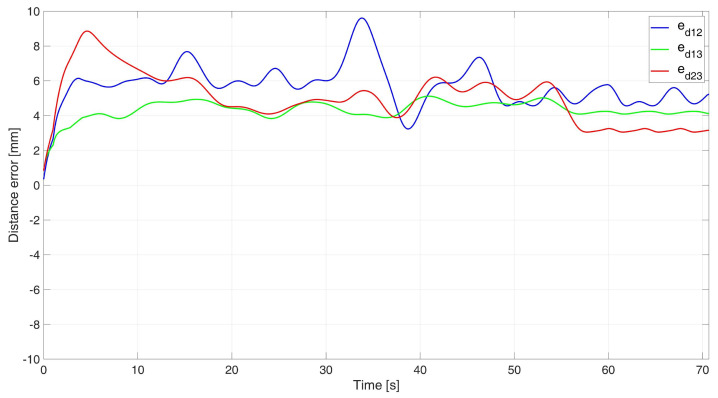
Distance errors between robots determined in the experimental study of synchronous motion with a group configuration controller.

**Figure 17:**
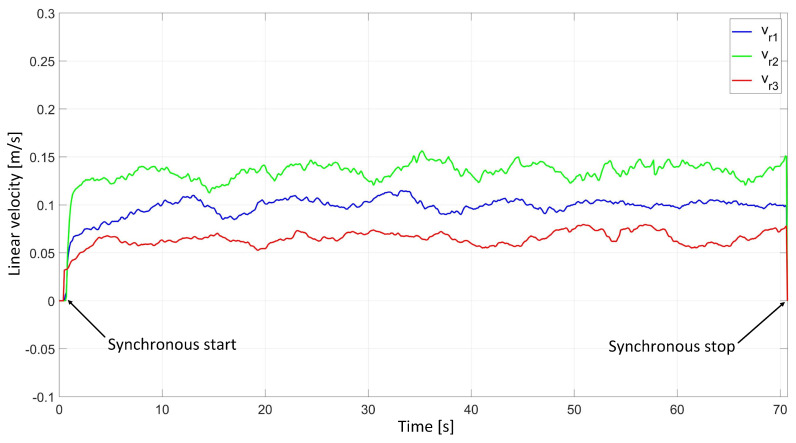
Linear velocities of robots determined in the experimental study of synchronous motion with a group configuration controller.

In the experimental study of the synchronous movement of a group along a given trajectory with the group configuration controller, the robots moved along a given trajectory with the declared group configuration, as can be seen in Figure 15. Analyzing the graphs of distance error (Figure 16), it can be seen that throughout the movement, the distance errors between the robots are minimized and maintained, and their values are acceptable and not greater than $e_{d12}$ = 9.6 mm, $e_{d13}$ = 5.1 mm, and $e_{d23}$ = 8.7 mm. The errors of trajectory tracking by individual robots in this study reached values no greater than $eR_{1}$ = 0.049 m, $eR_{2}$ = 0.036 m, and $eR_{3}$ = 0.037 m. Analyzing the velocity plot (Figure 17), it was noted that the robots started synchronously and completed the movement at the same time.

## 5. Conclusions

The use of mobile robots as transportation systems is a clear trend observed in industrial automation and robotization of production processes. Mobile robots are used on production lines to perform tasks such as the delivery of parts in the assembly process, receiving finished products, picking up full pallets of packed products, delivering empty pallets, and transporting goods in warehouses. However, the literature review conducted shows that, increasingly, robots are being integrated into groups to perform complex tasks. The huge potential of this issue is confirmed by a very large number of studies on control methods for groups of mobile robots. Considering the area of internal transportation, which is the area of interest in this work, attention was focused on methods dedicated to the control of groups with a decentralized structure. It was found that the main problem is time drift, which can even lead to interruption of communication between robots. Here, the possibility of improving the quality of decentralized group control by introducing time synchronization of units in the group in the algorithm was noted.

Based on the conducted research, it was found that the author’s proposed control algorithm using the modified consensus tracking method realizes synchronous movement of the group with preservation of the set shape of the formation even in the case of errors in communication between robots. The values of errors determined in the simulation studies are no larger than 5.5 mm for circular motion (compared to 55.8 mm for circular motion without including the group controller in the control algorithm). The errors determined in the experimental studies are acceptable from the point of view of using the robot group for transportation tasks.

The correct operation of the developed algorithm was confirmed during laboratory tests with real robots. The developed control method allows easy adaptation to commercially available AGV robots with a similar driving system and other controllers of trajectory tracking by individual robots.

## Data Availability

Dataset available on request from the authors.
